# On Intra-thoracic Solid Tumours

**Published:** 1845-10

**Authors:** 


*On Intra-thoracic Solid Tumours.*—The history of these morbid growths has hitherto attracted very little notice from any pathological writer. Dr. Gintrac of Bordeaux, twenty years ago, published a memoir on the diagnosis of various thoracic affections. Among these he mentions several instances of Steatomatous and Tuberculous tumours connected with the pleuræ; and now his son M. Henri Gintrac, following the footsteps of his father, has, in his recently published "Essai sur les Tumeurs Solides Intrathoraciques, 1845," collected together the histories of thirty-two cases of solid tumours—developed within the chest, but not appertaining to, or primarily connected with, the thoracic viscera. The structure of these growths or tumours was, as might be expected, very different in different cases; sometimes it was *encephaloid*, at other times *scirrhous*, and in a third set of cases it was *tuberculous*. In several instances it has been said to be of a *steatomatous* nature. We shall briefly relate a few cases of this rare affection.

*Case.*—A man, 75 years of age, had been for a length of time asthmatic, and much distressed with palpitation of the heart upon any exertion. He became dropsical, and gradually sunk and died.

On dissection, a circular, somewhat flattened, osseous tumour was found at the lower and inner part of the right lung, the substance of which remained perfectly sound, but somewhat compressed. The left lung also appeared quite healthy; but it had been pushed upwards and backwards against the vertebræ, the lower part of the pleural cavity being entirely occupied with a large tumour that had become developed there. It proved to be a cyst with hard bony parietes, adhering by its upper surface to the substance of the lung, and by its lower to the diaphragm. The question naturally suggests itself, how are we to account for the development of such tumours in the cavity of the chest? If we attempted to answer, we might be inclined to say—perhaps by the osseous transformation of false membranes that had been formed during an attack of pleuritis at some former period. Several cases are recorded by M. Gintrac that were analogous to the one now briefly related; and in the history of one or two, it is not difficult to follow the connection of the phenomena which seemed to trace back the formation of the intra-thoracic tumour to an old pleuritic attack.

In other cases, the development of the morbid growth had clearly nothing to do with any previous inflammation in the part; for it was of an encephaloid or scirrhous nature.

The situation of such tumours was sometimes between the pleura costalis and pleura pulmonalis; at other times, it was between the latter membrane and the substance of the lungs; or between the former and the ribs. Occasionally they occupied the place of the mediastina; and in a few rare cases they seemed to have originated in the osseous substance of the ribs themselves. Some of the cases of genuine intra-thoracic tumour have, at different times, been published as examples of degeneration or of hypertrophe of the thymus gland—an opinion which M. Gintrac considers to be quite erroneous.

It is rare that these tumours have been diagnosticated during the life of the patient. Occasionally indeed this has been the case, as in the following instance.

A man, who had for many years been subject to catarrhal complaints in the winter season, had an attack of pneumonia in 1830. In the end of May, 1839, he received a heavy blow on the lower and anterior part of the chest. On the following day he found two distinct swellings in the part that had been struck. Being examined at St. Andrew's hospital in Bordeaux, it was found that one of these swellings rested outwardly upon the cartilages of the third, fourth and fifth ribs, and inwardly upon the corresponding part of the fore surface of the sternum. It was irregular and knobby on the surface, resisting to the finger, without any sense of fluctuation, and firmly adherent to the subjacent parts. The second swelling, not more than four *centimetres* apart from the other, was larger, and of a hemispheric shape. It rested on the cartilages and anterior extremities of the eighth, ninth, tenth and eleventh right ribs, being situated externally, or rather farther back from the median line than the other; it was equally unyielding and immoveable.

The patient was suffering a good deal, at the time of his admission into the hospital, with dyspnœa; this became gradually worse and worse, and he died fifteen days afterwards.

From the external appearances, coupled with the existence of symptoms that indicated a compression of the lungs, M. Gintrac, senior, gave it, as his opinion, that there was an intra-thoracic tumour in this case.

On *dissection*, a large tumour was found to occupy the entire front of the mediastinum, adhering firmly to the posterior surface of the sternum, and the cartilages of the ribs. The heart was pushed back, and the lungs compressed against the vertebræ. Similar morbid growths were found attached to the right clavicle, and to the inner surface of the 10th and 11th ribs on the right side.

Now, even in this case, it is very doubtful whether any correct diagnosis could have been formed, if it had not been that the presence of the extra-thoracic tumour naturally enough suggested the idea of the co-existence of similar growths within the chest.

In a somewhat analogous case, MM. Corvisart and Leroux mistook an immense fibrous tumour, compressing the left lung, for an effusion of fluid into the pleura. The case is altogether so interesting, and represents so well the phenomena which usually accompany the existence of tumours within the cavity of the chest, that we are induced to give the particulars of it.

A man about 33 years of age, accustomed to hard labour, was subject to alternations of profuse perspirations and sudden chills; he was attacked with cough, accompanied with mucous expectoration; to which were soon added hoarseness and dyspnœa, along with a sensation of pricking and pain, extending from the throat to the extremity of the sternum, and which was sometimes so acute as to occasion fainting. The dyspnœa increased daily. Some months after, hemoptysis supervened; the pains augmented; the beatings of the heart became more frequent and tumultuous. The lower extremities became œdematous, and the sleep uneasy and agitated.

When he entered the hospital, his face was pale and puffy, and his eyelids were œdematous. A slight pain was felt towards the larynx and at the commencement of the trachea; the sputa were puriform, often tinged with blood; cough frequent; respiration short, deep and sighing. The left side of the chest was more *bombé*, and rounded than usual; on percussion it did not give out any sound. The right side gave an obscure sound anteriorly, and a more clear one on the back and side. On applying the hand over the heart, no movement or pulsation of that organ could be felt. The patient preferred the sitting posture, inclining forward; but he could lie down either on the back or on each side, though more frequently and with greater ease upon the left one. The sleep was troubled with painful dreams. The pulse in both arms was small, frequent, and tolerably regular; generally, a little less strong on the left than on the right side. The skin was slightly œdematous over the whole surface of the body: the feet were much engorged.

Corvisart, judging as much from the symptoms manifested since the attack of the disease, as from the signs furnished by the actual inspection of the patient, having especial regard to the total absence of sound in the left side of the chest, thought it was a case of effusion, filling all the left cavity of the thorax, and compressing the lungs so as to annihilate their functions. The breathing became worse, the expectoration altogether purulent, and the patient gradually sank and died.

*Dissection.*—In place of the effusion that had been predicted, there was a solid mass, of a reddish white colour, and of an irregular and nodulated surface, which filled the left side of the thorax, occupying also the usual place of the mediastinum, and extending upwards and in front on the right side. The left lung, of which the parenchyma was almost entirely disorganized, was reduced to a state that was almost lamellar: it contained besides an abscess in its substance. The right lung was considerably diminished in size; and the mediastinum, the pericardium, and the heart were pushed into the right cavity. The tumour was evidently situated between the debris of the upper lobe of the left lung, and the mediastinal pleura.

Pleural effusion is not the only morbid condition that may be mistaken for the presence of intra-thoracic tumours. M. Gintrac relates several cases where these growths have been accompanied with all the usual symptoms either of pulmonary or of cardiac disease. In a few instances, the most conspicuous symptoms were those indicative of hepatic derangement. With all our improved means of diagnosis, derived from Auscultation and Percussion, the case will almost always be one of perplexity and doubt. If there happen to be any outward tumours on or near to the thorax, and if the constitution of the patient be decidedly cachectic, and especially if the shape of the chest has become visibly altered since the first occurrence of the symptoms, we may possibly be led to form a correct opinion. The age of the patient may partly assist us; for most of the cases, hitherto observed, have occurred in middle-aged and old persons. In more than one instance, the aggravation, if not the invasion, of the symptoms was traceable to a blow or other injury of the chest. In almost all the cases collected together by our author, the heart was observed to be more or less displaced from its natural locality: this therefore is a phenomenon that deserves to be taken into account in our attempts to form a diagnosis. As a matter of course, the symptoms must vary alike in their character and intensity, according to the parts which are compressed or otherwise injured by the unnatural production within the thoracic cavity.—*Med. Chirurg. Review.*

